# The Role of High-Density Lipoproteins in Diabetes and Its Vascular Complications

**DOI:** 10.3390/ijms19061680

**Published:** 2018-06-05

**Authors:** Nathan K. P. Wong, Stephen J. Nicholls, Joanne T. M. Tan, Christina A. Bursill

**Affiliations:** 1 Immunobiology Research Group, The Heart Research Institute, 7 Eliza Street, Newtown, NSW 2042, Australia; Nathan.Wong@sahmri.com (N.K.P.W.); Joanne.Tan@sahmri.com (J.T.M.T.); 2 Discipline of Medicine, The University of Sydney School of Medicine, Camperdown, NSW 2006, Australia; 3 Heart Health Theme, South Australian Health and Medical Research Institute, North Terrace, Adelaide, SA 5000, Australia; Stephen.Nicholls@sahmri.com; 4 Adelaide Medical School, Faculty of Health & Medical Sciences, University of Adelaide, Adelaide, SA 5000, Australia

**Keywords:** diabetes mellitus, microvascular, macrovascular, complications, dyslipidaemia, high-density lipoprotein, apolipoprotein A-I, dysfunctional, atherosclerosis

## Abstract

Almost 600 million people are predicted to have diabetes mellitus (DM) by 2035. Diabetic patients suffer from increased rates of microvascular and macrovascular complications, associated with dyslipidaemia, impaired angiogenic responses to ischaemia, accelerated atherosclerosis, and inflammation. Despite recent treatment advances, many diabetic patients remain refractory to current approaches, highlighting the need for alternative agents. There is emerging evidence that high-density lipoproteins (HDL) are able to rescue diabetes-related vascular complications through diverse mechanisms. Such protective functions of HDL, however, can be rendered dysfunctional within the pathological milieu of DM, triggering the development of vascular complications. HDL-modifying therapies remain controversial as many have had limited benefits on cardiovascular risk, although more recent trials are showing promise. This review will discuss the latest data from epidemiological, clinical, and pre-clinical studies demonstrating various roles for HDL in diabetes and its vascular complications that have the potential to facilitate its successful translation.

## 1. Introduction

The growing prevalence of diabetes mellitus (DM) poses enormous health challenges worldwide and represents a significant burden of disease for communities across developed and developing nations. Latest figures from the World Health Organization estimate that 422 million adults were living with diabetes in 2014, with the global prevalence estimated at 8.5% of the adult population compared to only 4.7% in 1980 [1]. This figure is projected to rise to 592 million adults by 2035, with the bulk of the increase expected to be borne by low- and middle-income countries, in parallel with an increasing life expectancy, urbanisation, and associated lifestyle changes, such as obesity and physical inactivity [2,3].

Much of the burden of disease arises from a myriad of vascular complications that are strongly associated with DM, the pathophysiological hallmarks of which include inflammation, dysregulated angiogenesis, and atherosclerosis [4]. In particular, the accelerated development of atherosclerotic plaque in DM is a major contributor to macrovascular complications affecting multiple vascular beds, manifested as coronary artery disease (CAD), especially myocardial infarctions (MI), cerebrovascular disease, and peripheral arterial disease (PAD) [5]. In patients with type 1 (T1DM) or type 2 diabetes mellitus (T2DM), CAD represents the principal cause of death and there is an estimated two- to four-fold increase in mortality from CAD compared to non-diabetic individuals [6]. In addition, oxidative stress, chronic inflammation, and dysregulated angiogenesis are thought to play crucial roles and feature prominently in the development of microvascular complications, including chronic kidney disease (CKD), retinopathy, and neuropathy [5]. However, there is considerable overlap between these broad classifications. The risk of developing major PAD, for instance, is strongly associated with microvascular disease, particularly macroalbuminuria and previous retinal photocoagulation [7], while advanced imaging techniques have revealed the presence of early coronary microvascular dysfunction in T2DM patients without obstructive CAD [8]. These observations suggest common pathophysiological processes underlying a range of vascular complications.

While it is generally recognised that achieving intensive glycaemic control is an effective strategy to reduce the risk of microvascular complications, the findings of large-scale clinical studies in patients with T2DM suggest that such an intense strategy does not necessarily mitigate the risk of developing macrovascular complications and may, indeed, be harmful [9,10]. This contrasts with T1DM patients in whom intensive treatment aimed at achieving normoglycaemia can lead to long-term reductions in the risk of MI, stroke, and cardiovascular mortality [11]. Part of the explanation may relate to the clustering of several other risk factors with T2DM, such as hypertension, dyslipidaemia, and obesity, which is often collectively referred to as the metabolic syndrome [12].

The abnormal lipid profile that often accompanies T2DM is well-known to be associated with an increased risk of atherosclerotic vascular disease [13,14]. This “diabetic dyslipidaemia” is typically characterised by elevated serum triglycerides (TGs) and low high-density lipoprotein cholesterol (HDL) concentrations, together with raised apolipoprotein B and the prevalence of smaller, denser low-density lipoprotein (LDL) cholesterol particles [13,15]. In patients with T1DM, the degree of glycaemic control appears to be important, as those with poor control typically have dyslipidaemia resembling T2DM, while individuals with good control tend to have normal or even raised HDL [16]. Despite the success of lipid-modifying therapies, such as statins, in reducing adverse cardiovascular events, low HDL remains a significant and independent residual risk factor for the development of vascular complications associated with DM [17].

To date, a wealth of research indicates that HDL exerts diverse actions in the vascular system. It primarily mediates the process of reverse cholesterol transport (RCT) by scavenging cholesterol from peripheral cells, including from macrophages in atherosclerotic plaque, returning it to the liver for further metabolism and excretion [18]. In addition, HDL promotes endothelial function and has demonstrated anti-inflammatory, anti-oxidative, anti-thrombotic, and anti-diabetic properties, all of which should, conceivably, protect against vascular complications [19]. Unfortunately, however, many recent clinical studies evaluating HDL-raising therapies in patients with and without DM have not seen significant benefits to cardiovascular outcomes, indicating there is still much to learn about the biological functions of HDL [16].

This article aims to review recent evidence regarding the role of HDL in the pathophysiology and treatment of DM and its vascular complications. It will overview what is currently known about the molecular actions of HDL and examine the concept of “dysfunctional HDL” in DM.

## 2. Diabetic Complications and High-Density Lipoproteins (HDL) Levels

Ample epidemiological evidence consistently shows that low HDL levels are correlated with an increased risk of DM [20] and its vascular complications. In a multi-centre prospective study of almost 7000 patients, those with primary low levels of HDL (i.e., without abnormalities in TGs or LDL levels) were found to have double the prevalence of DM and a significantly higher risk of CAD compared to those with a normal lipid profile [21]. Similar associations have been reported regarding low HDL and an elevated incidence of stroke [22], especially in elderly patients with T2DM [23]. In patients with diabetic foot ulcers, a common manifestation of both PAD and peripheral neuropathy, low HDL levels were independent predictors of the incidence of lower extremity amputation and wound-related mortality [24,25]. With respect to microvascular disease, lower levels of HDL were, again, independently and positively correlated with the risk of developing diabetic nephropathy in patients with T1DM [26] and T2DM [27,28,29]. On the other hand, retinopathy in T2DM patients was not found to be significantly associated with serum HDL levels [29], indicating that HDL may not have a crucial role to play in this particular complication of DM.

Despite the consistent association of HDL levels with vascular complications in DM, it remains unclear as to whether a causal relationship exists between diabetes and reduced HDL, and, if so, the direction of such a relationship. It has been proposed that elevated plasma TGs, against a backdrop of insulin resistance, may initiate a mechanism by which HDL levels are reduced through increased catabolism and the actions of cholesteryl ester transfer protein (CETP), which facilitates the transfer of cholesteryl esters from HDL to TG-rich particles [30]. Conversely, low HDL levels may facilitate diabetes and its complications through the loss of protective functions on insulin-secreting pancreatic beta cells [31] and endothelial cells [32]. It is possible that both mechanisms play a role, with diabetes and dyslipidaemia forming a vicious cycle leading to vascular complications.

Yet, conflicting genetic studies have also cast uncertainty on the relationship between HDL, DM, and vascular complications. While some suggest that a genetic predisposition to low HDL predicts an elevated risk of T2DM [33], others, using a Mendelian randomization approach, have found that genetic variants corresponding to low HDL did not associate with an increased risk of T2DM [34]. Moreover, analysis of a range of single-nucleotide polymorphisms (SNPs) linked to increased plasma HDL levels have revealed that these do not predict a lower risk of MI [35] nor the risk of CAD more broadly [36]. In particular, the Taq1B polymorphism in the *CETP* gene has been widely investigated as it is recognised to modulate plasma HDL concentrations, including in patients with T2DM [37]. Some studies have found that genetically lower CETP concentrations and lower CETP activity associated with this polymorphism can lead to a lower risk of CAD [38,39], while other studies have not replicated this [40]. Indeed, concurrent effects on non-HDL lipid parameters, such as LDL and TGs, are not excluded. Such inconsistency highlights that a causal role for HDL in cardiovascular disease remains equivocal from a genetic standpoint, though there is a lack of large-scale genetic studies that have specifically addressed the issue of HDL functionality, which may be a more important determinant of cardiovascular outcomes.

## 3. Molecular Functions of HDL in Diabetes

HDLs are complex particles comprised of a hydrophobic lipid core, containing TGs and esterified and non-esterified cholesterol, surrounded by an outer layer of phospholipids and proteins (termed apolipoproteins), the most abundant of which is apolipoprotein A-I (apoA-I).

### 3.1. HDL and Glucose Metabolism

**Figure 1:**
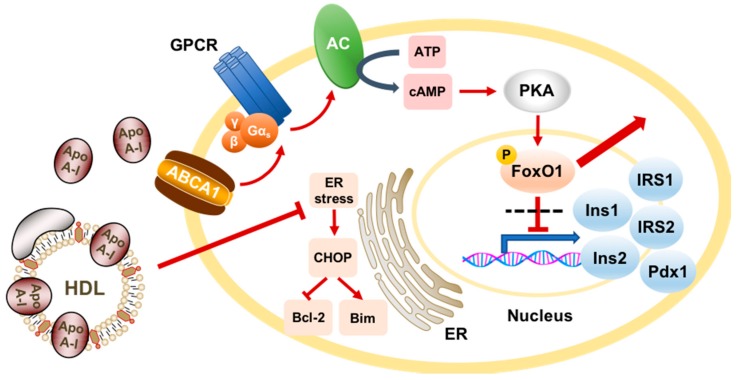
Schematic representation of the actions of apolipoprotein A-I (apoA-I) and high-density lipoprotein (HDL) in pancreatic beta cells. ApoA-I binds to the adenosine triphosphate (ATP)-binding cassette transporter A1 (ABCA1) and induces its colocalisation with the Gα_s_ subunit of a G-protein-coupled receptor (GPCR). This activates adenylate cyclase (AC) to convert ATP to cyclic adenosine monophosphate (cAMP), which in turn activates protein kinase A (PKA). Phosphorylation of the transcription factor forkhead box protein O1 (FoxO1) by PKA induces its exclusion from the nucleus, which derepresses the transcription of genes involved in insulin secretion and beta cell survival, such as insulin 1 (Ins1) and 2 (Ins2), insulin receptor substrate 1 (*IRS1*) and *2* (*IRS2*), and *Pdx1*. HDL also exerts anti-apoptotic effects in pancreatic beta cells by counteracting the endoplasmic reticulum (ER) stress response under conditions of high glucose. This response to prolonged ER stress would usually lead to stimulation of the apoptotic mediator CHOP, which decreases anti-apoptotic factors, such as Bcl-2, and increases pro-apoptotic factors, such as Bim. Red arrows denote a stimulatory effect while T bars denote an inhibitory effect.

HDL has been demonstrated to mediate a range of beneficial actions in diabetes. An emerging body of evidence suggests a direct role for HDL in glycaemic control through its actions on pancreatic beta cells (Figure 1). This has been the subject of previous comprehensive reviews [31,41]. Treatment of Min6 beta cells and isolated pancreatic islets from rats with lipid-free apoA-I or reconstituted HDL (made by complexing apoA-I with phospholipid 1-palmitoyl-2-linoleoylphosphatidylcholine, PLPC) stimulated increased insulin production and secretion under both basal and high-glucose conditions [42]. The effects of apoA-I were dependent on the classical HDL receptor, scavenger receptor class B type I (SR-BI), as well as the adenosine triphosphate-binding cassette (ABC) transporter A1 (ABCA1), which exports cholesterol and phospholipids from cell membranes to apoA-I forming nascent HDL. Mechanistically, apoA-I induces colocalisation of ABCA1 with the α_s_ subunit of a G-protein coupled receptor, activating the cyclic adenosine monophosphate (cAMP)–protein kinase A (PKA) pathway and promoting exclusion of the key transcription factor forkhead box protein O1 (FoxO1) from the nucleus. This leads to the upregulation of genes involved in insulin secretion and beta cell survival, including insulin 1 and 2, insulin receptor substrate (*IRS*) 1 and *2*, and *Pdx1* [43].

Consistent with this, apoA-I and HDL have been shown to inhibit glucose- and interleukin-1β-induced apoptosis in human and murine pancreatic islets [44]. Such a protective function for HDL may be related to its capacity to counteract the endoplasmic reticulum (ER) stress response in pancreatic beta cells by dampening ER stress signalling, reversing disruptions to ER morphology, and augmenting normal protein folding and export mechanisms [45]. However, this may not be the only pathway, as HDL has also been demonstrated to inhibit apoptosis induced by the protein glycosylation inhibitor, tunicamycin, in Min6 cells without altering the ER stress response or ER function [46]. This redundancy potentially highlights the ability of HDL to defend beta cells against a range of cellular insults.

Several in vivo studies further support the beneficial role of apoA-I and HDL in regulating glucose metabolism. A genetic knockout study of *apoA-I* in mice showed a phenotype of significantly impaired glucose tolerance compared to wild-type mice [47]. Moreover, insulin-resistant mice injected with a single intraperitoneal dose of human apoA-I showed acutely increased insulin secretion and capacity for glucose clearance in response to a glucose tolerance test [48]. In a study of human patients with T2DM, intravenous infusions of rHDL over four hours were found to reduce plasma glucose levels by increasing insulin secretion and stimulating glucose uptake into skeletal muscle via activation of the 5′ AMP-activated protein kinase (AMPK) pathway through ABCA1 [49]. Such studies suggest that the anti-diabetic actions of HDL hold great promise if they can be harnessed for therapeutic benefit.

### 3.2. HDL and Atherosclerosis in Diabetes

**Figure 2:**
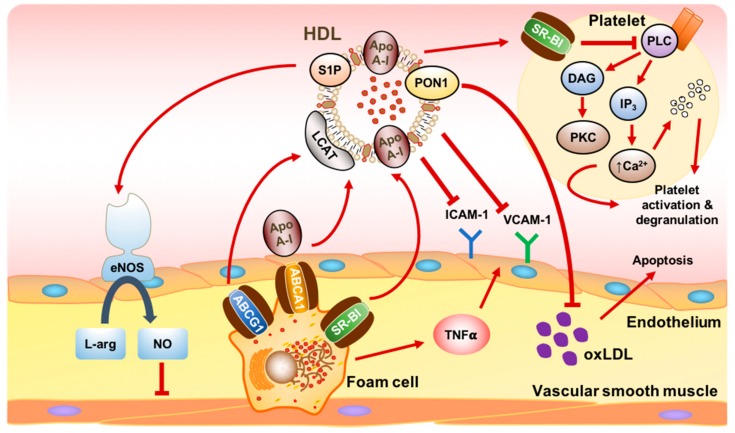
Schematic representation of the diverse actions of HDL in atherosclerosis. HDL mediates reverse cholesterol transport by scavenging cholesterol from lipid-laden macrophages (foam cells) in atherosclerotic plaque. ApoA-I initiates cholesterol efflux by binding to ABCA1, with further uptake occurring via the ATP-binding cassette transporter G1 (ABCG1) and scavenger receptor class B type I (SR-BI). Cholesterol is esterified by lecithin-cholesterol acyl transferase (LCAT) and incorporated into the lipid core of mature HDL prior to transport to the liver for excretion. HDL also promotes endothelial function by stimulating endothelial nitric oxide synthase (eNOS) through the bioactive lipid shingosine-1-phosphate (S1P). eNOS converts l-arginine (l-arg) to nitric oxide (NO), which decreases vascular smooth muscle tone and reactivity. HDL exerts anti-apoptotic effects through paraoxonase-1 (PON-1), which hydrolyses oxidised LDL (oxLDL), a potent stimulator of apoptosis. HDL also inhibits the expression of recruitment factors for inflammatory cells, such as the intercellular adhesion molecule-1 (ICAM-1) and vascular cell adhesion molecule (VCAM-1), which are stimulated in response to tumour necrosis factor α (TNFα) released by foam cells. Platelet activation is reduced by HDL through its interactions with SR-BI. HDL inhibits phospholipase C (PLC)-mediated production of diacylglycerol (DAG) and inositol trisphophate (IP_3_), which reduces protein kinase C (PKC) activation, intracellular calcium (Ca^2+^) mobilisation, and subsequent platelet degranulation. Red arrows denote a stimulatory effect while T bars denote an inhibitory effect.

HDL also confers benefits far beyond merely affecting glucose homeostasis in diabetes. HDL has well-established roles in RCT and the maintenance of endothelial function, and in doing so, protects against atherosclerosis, a pathological hallmark of vascular complications [19] (Figure 2). HDL achieves this by mediating cholesterol efflux from lipid-rich macrophages in the arterial walls of atherosclerotic plaque, firstly, via interactions of apoA-I with ABCA1, then, via further uptake through SR-BI and the ABC transporter G1 (ABCG1) [50]. The cholesterol is esterified by lecithin-cholesterol acyl transferase (LCAT), which stabilises it in the lipid core, allowing it to be transported to the liver for metabolism or biliary excretion, or alternatively, transferred to TG-rich lipoproteins through the actions of CETP [18]. This process of cholesterol removal reduces the lipid burden within atherosclerotic plaque and characterises the atheroprotective role of HDL.

The ability of HDL to induce plaque regression and stabilisation has been demonstrated in various in vivo studies. Adenoviral gene transfer of human apoA-I to LDL receptor-deficient mice was found to significantly increase HDL concentrations and induce regression of aortic fatty streak lesions [51]. Other studies have used apoA-I Milano (apoA-I_M_), a variant form of apoA-I associated with protection from atherosclerosis, despite very low HDL concentrations [52]. In apolipoprotein E-deficient mice, infusions of rHDL made from recombinant apoA-I_M_ and phospholipid prevented the progression of aortic plaques, with a concomitant reduction in their lipid and macrophage content [53], an effect that was replicated as early as 48 h following a single high dose [54]. Likewise, in rabbit models of atherosclerosis, infusions of a commercial apoA-I_M_/phospholipid complex, ETC-216, have been shown to induce rapid plaque regression based on assessment by intravascular ultrasound (IVUS) or magnetic resonance imaging [55,56]. Moreover, in a landmark trial of human patients with recent ACS, five weekly infusions of ETC-216 led to a small, though significant, regression of coronary atheromas using IVUS [57]. Although these studies were not specifically done in the context of DM, they, nevertheless, highlight a beneficial role for HDL in atherosclerotic vascular disease.

In endothelial cells (ECs), HDL promotes endothelial homeostasis by stimulating endothelial nitric oxide synthase (eNOS) to produce nitric oxide (NO), a potent mediator of vascular relaxation and an inhibitor of platelet aggregation and leukocyte adhesion [58]. Administration of rHDL to T2DM patients significantly reversed the baseline reduction in endothelial NO availability [59]. One important mechanism in vivo involves sphingosine-1-phosphate (S1P), a bioactive phospholipid carried by HDL that can activate eNOS and helps to maintain the integrity of the endothelial barrier [58]. In a mouse model of T1DM, S1P was also shown to prevent monocyte-endothelial interactions by activating the S1P1 receptor to inhibit downstream inflammatory pathways [60]. S1P is typically reduced in HDL derived from patients with DM [61]. HDL can also directly enhance the survival of ECs by inhibiting apoptosis due to oxidised LDL or inflammatory cytokines, such as tumour necrosis factor alpha (TNFα). The anti-apoptotic response may be related to reduced activation of the ER stress response [62], as well as inhibition of intracellular reactive oxygen species (ROS), thereby, preventing cytochrome *c* release from mitochondria and activation of the caspase cascade [63]. Yet, HDL is also able to directly counteract the oxidation of LDL through its anti-oxidant effects. This is achieved, at least in part, by its association with enzymes, such as paraoxonase-1 (PON-1), which serves to hydrolyse oxidised lipids [18] and whose activity is inversely correlated with the risk and severity of CAD in T2DM patients [64].

In addition, HDL has potent anti-inflammatory effects in the vasculature, particularly through the inhibition of intercellular adhesion molecule-1 (ICAM-1), vascular cell adhesion molecule-1 (VCAM-1), and monocyte chemoattractant protein-1 (MCP-1). These are induced by inflammatory cytokines and lead to the recruitment and infiltration of monocytes into arterial walls, representing an initiating step in atherosclerosis [50]. Infusion of rHDL in T2DM patients augmented the anti-inflammatory properties of plasma HDL from these patients, which were better able to suppress the expression of ICAM-1 and VCAM-1 from ECs in a concentration-dependent manner [65].

Furthermore, platelets from diabetic patients have increased reactivity and baseline activation, predisposing them to thrombotic complications, such as atherosclerotic plaque rupture, as occurs with MIs [66]. The treatment of platelets in vitro with rHDL resulted in attenuated aggregation and adhesion responses, and suppressed thrombus formation in flow-based assays over a collagen matrix [67]. Similarly, short-term infusions of rHDL in patients with T2DM were effective at inhibiting ex vivo platelet aggregation, likely related to cholesterol depletion from platelet membranes [67]. These anti-thrombotic effects of HDL may be mediated by SR-BI receptors on platelets through a mechanism involving cleavage of diacylglycerol (DAG) by phospholipase C and subsequent activation of protein kinase C, ultimately, inhibiting platelet activation [68].

### 3.3. HDL and Angiogenesis in Diabetes

**Figure 3:**
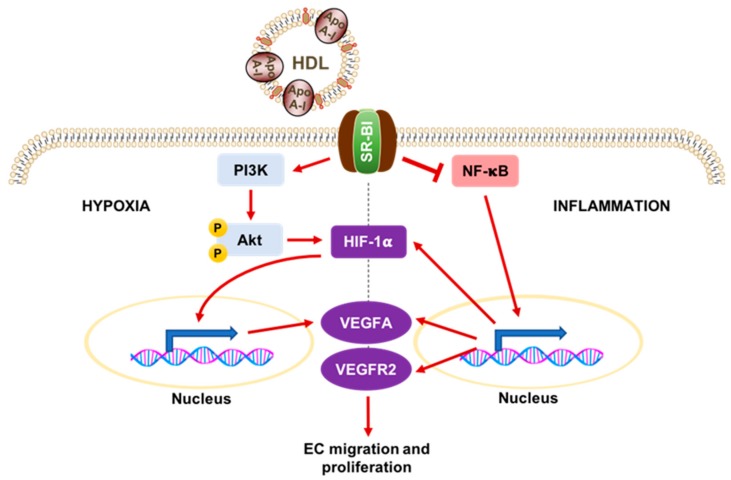
Schematic representation of HDL and its context-dependent regulation of angiogenesis. In hypoxia, HDL activates phosphatidylinositol-3-kinase (PI3K) via SR-BI, leading to phosphorylation of Akt and activation of the transcription factor hypoxia-inducible factor-1α (HIF-1α). This translocates to the nucleus where it increases expression of the vascular endothelial growth factor (VEGF) and VEGF receptor 2 (VEGFR2). These promote endothelial cell migration and proliferation, which are crucial steps in the physiological angiogenic response to hypoxia. Conversely, in inflammation, HDL reduces the activation of the key transcription factor, nuclear factor kappa B (NF-κB), suppressing the expression of HIF-1α, VEGFA, and VEGFR2. This reduces pathological inflammatory-driven angiogenesis. Red arrows denote a stimulatory effect while the T bar denotes an inhibitory effect.

Many vascular complications in diabetes are also characterised by dysregulated angiogenesis, the process by which new blood vessels are formed from the existing vasculature. Angiogenesis underpins the normal process of wound healing and is crucial in the formation of collateral vessels as an adaptive response to ischaemia, yet it is also involved in driving pathological inflammatory processes, such as atherosclerosis [4]. Early evidence for the role of HDL in angiogenesis came from Sumi et al. [69] who showed that rHDL promoted the in vitro differentiation of endothelial progenitor cells (EPCs) via the phosphatidylinositol 3-kinase (PI3K)/Akt pathway. Subsequent infusions of rHDL in a murine model of hindlimb ischaemia resulted in mobilisation of bone-marrow derived EPCs to the ischaemic limb, culminating in improved blood flow recovery [69]. In line with this, infusion of rHDL in seven patients with T2DM found that circulating EPCs were increased one week post administration [70]. More recent evidence also indicates that HDL can regulate angiogenesis in a context-dependent manner (Figure 3). Studies from cultured human ECs [71] and murine models [72] show that apoA-I/rHDL enhances physiological angiogenesis in response to hypoxia, but suppresses pathological inflammatory angiogenesis. HDL achieves this by activating distinct cellular signalling pathways downstream of SR-BI, modulating the expression of key angiogenic mediators, such as hypoxia-inducible factor-1α (HIF-1α) and vascular endothelial growth factor (VEGF), with subsequent effects on EC migration and proliferation [73]. Diabetic vascular complications are associated with an impairment of ischaemia-induced neovascularisation [74]. rHDL was found to rescue diabetes-impaired angiogenesis by enhancement of key signalling mediators in the hypoxia-driven angiogenic axis, enhancing post-translational HIF-1α modulation and nuclear translocation, increasing VEGFA/VEGFR2 production and signalling, and augmenting eNOS activation [75]. These effects were all dependent on SR-BI. Similarly, in diabetic mice, infusion of rHDL reversed the diabetes-induced impairment of angiogenesis following hindlimb ischaemia, while diabetic wounds treated with topical rHDL showed an improved healing capacity and wound angiogenesis [75]. These effects were abrogated in *SR-BI* knockout mice, suggesting a crucial role for SR-BI in the modulation of angiogenesis in DM by HDL.

The recognition that HDL mediates such a diverse range of protective functions has made it a promising target for therapies designed to ameliorate diabetic vascular complications.

## 4. Dysfunctional HDL in Diabetes

In recent years, much of the research focus has shifted from attempts to optimise HDL levels towards understanding broader aspects of HDL composition and function, as “dysfunctional HDL” is recognised to play an increasingly critical role in diabetes and its complications [50,76,77].

In fact, the HDL plasma fraction is known to comprise a highly heterogeneous group of particles, with varying sizes, densities, and compositions [78]. There is evidence from epidemiological studies that these can be altered in the setting of diabetes towards a phenotype favouring HDL dysfunction and vascular complications. For instance, the levels of HDL2 particles, which are relatively larger, cholesterol-rich, and less dense, are inversely associated with the incidence of T2DM [79,80]. This has implications for the development of vascular complications, as previous studies have reported that HDL particle size and the distribution of HDL sub-classes were significantly altered in patients with CAD complicated by DM, compared to those with CAD, but not DM [81]. In particular, the former group was found to have higher levels of smaller, denser HDL3 particles and lower HDL2. Similarly, in men with T2DM, the prevalence of smaller, denser, apoA-I-depleted HDL was associated with increased risks of CAD, microvascular complications, and impaired pancreatic beta cell function [82].

In the setting of DM and atherosclerosis, the diverse range of biological functions mediated by HDL can be deleteriously altered [83]. RCT can be impaired under high-glucose conditions in vitro, as well as in diabetic animal models, through the downregulation of transporters such as ABCA1 and ABCG1 in macrophages, which is potentially mediated by ROS and/or advanced glycation end products [84]. Indeed, macrophages from patients with T2DM have been found to have reduced ABCG1 expression, leading to increased cholesterol accumulation [85]. Meanwhile, high glucose conditions stimulate SR-BI expression in macrophages, but, surprisingly, this leads to a switch from HDL-mediated cholesterol efflux to cholesterol influx [86], thus, providing a putative mechanism for accelerated atherosclerosis in DM. There are conflicting results, however, as one study showed that non-enzymatic glycation of apoA-I, as occurs in vivo in DM, reduces its affinity for phospholipids, but had no effect on ABCA1- or ABCG1-dependent cholesterol efflux from lipid-laden macrophages [87]. This suggests there is still more to learn about HDL and its dysfunctional characteristics in RCT.

There is evidence that other crucial functions of HDL, such as endothelial protection and repair, are also adversely modified by diabetes, most likely through the alteration of specific components of HDL particles. HDL isolated from T2DM patients have an impaired ability to stimulate eNOS activity [88] and NO production from ECs [89] compared to HDL from healthy controls, and this was found to be related to a drop in plasma S1P levels [88]. In contrast, other studies have reported a significant increase in HDL-associated S1P in patients with T2DM, leading to an increased expression of endothelial protective mediators, such as cyclooxygenase-2 and prostacyclin I-2 [90]. Interestingly, this rise in S1P was reversed in T2DM patients who had developed macrovascular atherosclerosis, suggesting that increases in HDL-bound S1P may be an early compensatory mechanism that may be lost with T2DM progression. Indeed, S1P enrichment of glycated HDL in vitro was able to restore its endothelial protective functions [91]. These studies suggest that S1P signalling has a role in diabetic complications and further elucidation may identify important therapeutic targets [92].

HDL from T2DM patients also exhibit an impaired capacity to stimulate EC migration, proliferation, and extracellular matrix adhesion, owing to diabetes-induced downregulation of SR-BI and an impaired ability to maintain Akt activation [93]. Interestingly, plasma HDLs from normal controls that were modified by oxidation or glycation in vitro demonstrated similar dysfunctional effects to diabetic HDL, suggesting that such modifications may be biologically important in accounting for HDL dysfunction [93]. Oxidised HDL also impairs the function of EPCs in vitro, increasing apoptosis and intracellular ROS levels, leading to reduced EPC migration and angiogenesis through a pathway involving mitogen-activated protein kinase (MAPK) and nuclear factor kappa B (NF-κB) [94].

One of the crucial targets of oxidation and glycation in vivo may be apoA-I. In serum from T2DM patients, apoA-I has been shown to be specifically targeted for oxidation by myeloperoxidase (MPO), a potent oxidative enzyme, leading to impairment of its anti-apoptotic activity in ECs [95]. Selective oxidation of apoA-I at specific sites by MPO occurs frequently in human atherosclerotic plaque, with resulting profound functional impairment of apoA-I with regard to ABCA1-dependent cholesterol efflux [96,97]. Injections of *apoA-I*-knockout mice with isolated human apoA-I oxidised by MPO ex vivo significantly impaired RCT in vivo compared to injections of native human apoA-I [98]. Oxidative modifications of apoA-I also suppress the anti-inflammatory activities of HDL on ECs in vitro, while inducing a gain of pro-inflammatory functions [99]. Glycation of apoA-I, as a result of exposure to hyperglycaemia, also impaired its ability to reduce VCAM-1 and ICAM-1 expression in a rabbit peri-arterial cuff model [100]. Accordingly, HDL from DM patients was unable to inhibit TNFα-induced activation of NF-κB and phosphorylation of the key pro-inflammatory transcription factor, p65, in ECs, as opposed to HDL from normoglycaemic controls [88]. Moreover, HDL from T2DM patients had an impaired ability to inhibit LDL oxidation and LDL-induced monocyte chemotaxis [101].

Dynamic alterations to the proteomic and lipidomic composition of HDL can also occur in those with DM. HDL from patients with DM is relatively enriched with proteins, such as the acute phase reactant serum amyloid A (SAA), apolipoprotein C-III, and MPO, which tend to confer pro-oxidative, pro-inflammatory, and impaired cholesterol efflux properties to HDL [50,77,102,103]. Conversely, anti-oxidative and anti-inflammatory proteins, such as apoA-I, PON-1, PON-3 and platelet-activating factor-acetylhydrolase (PAF-AH), are relatively lost, resulting in a shift from an anti-atherogenic to a more dysfunctional pro-atherogenic phenotype [50,76,77]. Post-translational modifications of these HDL-associated proteins and lipids play a role in diabetic vascular complications. The extent of apoA-I glycation and reduction in PON-1 activity was positively correlated with the presence and angiographic severity of CAD in T2DM patients [64]. In addition, HDL from patients with T2DM was found to have higher levels of oxidised fatty acids relative to non-diabetic controls, an observation that was even more pronounced in those with concurrent T2DM and CAD [104].

There is also strong evidence to suggest that similar mechanisms of HDL dysfunction exist in patients with T1DM [105]. Despite having normal or elevated HDL concentrations, T1DM patients with good glycaemic control, nevertheless, have an increased risk of adverse cardiovascular events compared to age-matched non-diabetics [106]. A recent study found that children and young adults with T1DM have reduced HDL function as measured by its ability to release lipid-poor apoA-I [107]. Similarly, the ex vivo cholesterol efflux capacity of HDL isolated from patients was significantly lower relative to non-diabetic individuals and was associated with more abundant oxidative modifications of several apolipoproteins [108]. Moreover, HDL particles from patients with T1DM have dysfunctional anti-oxidative actions and this is particularly pronounced in those with coexistent microalbuminuria [109]. Changes in HDL composition also appear to play a role. There is increased pro-inflammatory SAA associated with HDL2 and HDL3 particles in patients with T1DM, especially in the context of poor glycaemic control [103], together with significant modifications of the phosphosphingolipid profile, in particular, reduced S1P in total plasma and HDL2 of patients with T1DM [110].

Taken as a whole, it is clear that a renewed focus on understanding the function, rather than quantity, of HDL is justified, and future therapies seeking to manage vascular complications in DM will need to address the issue of overcoming HDL dysfunction.

## 5. HDL-Modifying Treatment Approaches in Diabetes

Currently, statins are the mainstay treatment for diabetic dyslipidaemia. These drugs inhibit the 3-hydroxy-3-methylglutaryl-coenzyme A (HMG-CoA) reductase, a rate-limiting enzyme involved in cholesterol biosynthesis, primarily resulting in lower LDL levels, but also modest reductions in TGs and increases in HDL and apoA-I [15,111]. Pitavastatin was shown to increase cholesterol efflux from THP-1 macrophages in vitro, as well as enhancing HDL’s anti-oxidative function by increasing PON-1 [112], though this conflicts with studies showing that statin therapy reduces ABCA1-mediated cholesterol efflux to HDL [113]. Nevertheless, a meta-analysis of statin therapy in over 18,000 patients with T2DM showed significant reductions in cardiovascular events, such as MI and stroke, along with reduced mortality rates [114]. Similar effects were seen in a smaller population of patients with T1DM [114]. More recently, high-intensity statin therapy was also shown to induce coronary atherosclerotic plaque regression in both diabetic and non-diabetic individuals [115]. However, concerns have been raised that statins increase the risk of developing T2DM, especially in patients at higher risk, such as those with metabolic syndrome [116]. This has been confirmed in several meta-analyses [117,118], most recently one that included 20 observational studies and found a class effect of statins, increasing the risk of incident DM [119]. The mechanisms underlying this diabetogenic effect remain unclear, though adverse effects on pancreatic beta-cell function and insulin sensitivity have been implicated, as well as genetic polymorphisms in *HMG-CoA* [116]. Despite this, the risk of incident DM with statins appears only modest and is outweighed by the benefits of cardiovascular risk reduction [120].

Ezetimibe, an inhibitor of intestinal cholesterol absorption via the NPC1L1 transporter, has also been investigated as a supplement to statin therapy, especially in those with inadequately controlled LDL levels. Compared to using statins alone or, indeed, doubling of the statin dose, adding ezetimibe to a statin confers significantly larger reductions in LDL [121,122,123] and leads to a reduced incidence of major adverse cardiac events in patients with DM [124]. The effects on HDL are less clear as some studies have reported that ezetimibe augments the increase in HDL levels achieved with statins [122,125], while others have observed a decrease in HDL secondary to ezetimibe [126]. Nevertheless, the IMPROVE-IT study recently showed that the addition of ezetimibe to simvastatin significantly improved cardiovascular outcomes compared to simvastatin alone in high-risk patients following acute coronary syndromes (ACS) [127], with a subgroup analysis finding that these benefits were particularly pronounced in patients with DM compared to those without DM [128]. Indeed, in a rat model of T2DM, chronic ezetimibe treatment was found to increase pancreatic beta-cell mass and the activity of glucagon-like peptide-1, an incretin hormone that stimulates insulin release, providing some mechanistic insight into the benefits of ezetimibe in DM [129].

More recently, monoclonal antibodies that inhibit proprotein convertase subtilsin/kexin type 9 (PCSK9) have garnered much interest in the treatment of diabetic dyslipidaemia. These agents inhibit the binding of PCSK9 to LDL receptors, which targets them for lysosomal degradation. The currently available PCSK9 inhibitors, alirocumab and evolocumab, reduce LDL cholesterol to a greater degree than statins, typically by 50–60%, while also modestly elevating HDL by 7–11% [130]. These effects appear to be similar in patients with or without T2DM [131]. The FOURIER trial was the first to show improved cardiovascular outcomes with evolocumab in statin-treated patients with atherosclerotic vascular disease [132], with a subgroup analysis showing comparable reductions in cardiovascular risk in diabetic patients relative to non-diabetic patients [133]. Recent randomized trials of alirocumab also found superior LDL reductions when compared to a placebo in patients with insulin-dependent T1DM and T2DM on maximal statin therapy [134], as well as compared to standard lipid-lowering therapies in patients with T2DM [135]. Despite this, recent Mendelian randomization studies found that *PCSK9* genetic variants associated with low LDL also predict an increased risk of new-onset DM [136,137]. Fortunately, this effect has not been borne out in clinical trials [133,138,139], with an overall efficacy and safety profile that makes PCSK9 inhibitors very promising as future therapeutic agents.

Several HDL-raising agents have also been trialled recently in patients with DM, though with generally disappointing results [140,141]. Extended-release niacin raises HDL levels by up to 35%, while also reducing TGs and LDL [141]. In patients with T2DM, niacin therapy rescued the impaired capacity of diabetic HDL to stimulate endothelial NO production, combat oxidative stress, and promote endothelial repair via EPCs [89]. However, results from two large-scale clinical studies, AIM-HIGH [142] and HPS2-THRIVE [143], showed no significant benefit of adding niacin to statin therapy, with respect to the risk of major adverse cardiovascular events in both diabetic and non-diabetic patients. Indeed, while niacin effectively raised HDL concentrations, it did not have any impact on the cholesterol efflux capacity or anti-inflammatory functions of HDL [144], perhaps related to a mechanism of statin interference [77]. Moreover, niacin has been associated with well-recognised flushing side-effects and an increased risk of new-onset DM that has limited its clinical use [145].

Clinical trials of the CETP inhibitors, torcetrapib [146], dalcetrapib [147], and evacetrapib [148], also did not demonstrate significant benefits to CAD outcomes in either diabetic or non-diabetic patients, in spite of their ability to raise HDL levels. In fact, torcetrapib caused significant harm, increasing the rate of cardiovascular events and all-cause mortality [146]. More recently, however, the REVEAL study of anacetrapib in statin-treated patients was able to show, for the first time, a reduced incidence of major CAD complications concomitant with higher HDL and lower non-HDL levels, along with a reduced occurrence of new-onset DM relative to patients treated with a placebo [149]. Indeed, HDL isolated from anacetrapib-treated patients was previously shown to augment cholesterol efflux from foam cells and to have preserved anti-inflammatory properties via ABCA1- and ABCG1-dependent pathways [150]. Despite this, it has been suggested that the positive effects of anacetrapib may be exerted more through the reduction of non-HDL cholesterol rather than the elevation of HDL [151].

Given some of the variable effects of the above drugs, there has also been mounting interest in the development of HDL analogues and apoA-I mimetic peptides for the treatment of vascular diseases [140,152]. Studies of such novel compounds have, so far, mainly been confined to in vitro studies or animal models, however, several do show promise. In a murine model of diabetic atherosclerosis, for instance, treatment with the apoA-I mimetic, D-4F, resulted in a significantly reduced atherosclerotic lesion area and macrophage infiltration [153]. Further assessment of similar agents in clinical studies involving diabetic patients are eagerly awaited.

In recent years, there has also been growing interest in the role of microRNAs (miRNAs) in the pathophysiology of diabetic vascular complications [154,155]. miRNAs consist of short (about 21–24 nucleotides), non-coding, single-stranded RNA molecules that post-transcriptionally regulate target gene mRNAs by specifically base-pairing to their 3′ untranslated regions (UTR), thereby, inhibiting translation and promoting their degradation [156]. Various miRNAs have been linked to vascular complications in DM through effects on diverse cell types and functions [154,155], and these are proposed to be promising therapeutic targets [157] or biomarkers [158]. Indeed, there is evidence that HDL and other lipoproteins can act as carriers of miRNAs, delivering these to target cells [159,160]. The function and metabolism of HDL itself can be regulated by miRNAs, in particular miR-33a and miR-33b, which have putative roles in cholesterol efflux and HDL synthesis [161]. Whether miRNAs mediate the actions of HDL in diabetes and its vascular complications remains to be investigated.

## 6. Conclusions

In summary, the rising prevalence and burden of DM worldwide demands the development of novel therapies to tackle a broad range of associated complications. HDL continues to represent a promising therapeutic target due to its myriad of protective functions encompassing reverse cholesterol transport, glucose homeostasis, and endothelial function, as well as anti-inflammatory, anti-oxidative, anti-thrombotic, anti-apoptotic, and pro-angiogenic properties. Moving forward, however, it is evident that new therapies designed to target HDL in DM will need to overcome numerous pathologically altered aspects of its function. Beyond their effects on HDL levels, relatively little is known at present about the ability of current treatments to modify HDL dysfunction and this may explain their relative lack of efficacy in clinical trials. Further delineation of the factors driving HDL function may help in identifying potential novel biomarkers or parameters to stratify risk and monitor treatment response, as well as facilitate the translation of HDL-modifying therapies to clinical practice.
